# Impact of Dental Pulp Stem Cells Overexpressing Hepatocyte Growth Factor after Cerebral Ischemia/Reperfusion in Rats

**DOI:** 10.1016/j.omtm.2018.07.009

**Published:** 2018-08-04

**Authors:** Kota Sowa, Chikako Nito, Masataka Nakajima, Satoshi Suda, Yasuhiro Nishiyama, Yuki Sakamoto, Yuko Nitahara-Kasahara, Aki Nakamura-Takahashi, Masayuki Ueda, Kazumi Kimura, Takashi Okada

**Affiliations:** 1 Department of Neurological Science, Graduate School of Medicine, Nippon Medical School, Tokyo 113-8603, Japan; 2 Department of Biochemistry and Molecular Biology, Graduate School of Medicine, Nippon Medical School, Tokyo 113-8603, Japan; 3 Department of Cell and Gene Therapy, Graduate School of Medicine, Nippon Medical School, Tokyo 113-8603, Japan; 4 Department of Pharmacology, Tokyo Dental College, Tokyo 101-0061, Japan; 5 Department of Neurology and Stroke Medicine, Tokyo Metropolitan Tama Medical Center, Tokyo 183-8524, Japan

**Keywords:** focal cerebral ischemia, hepatocyte growth factor, dental pulp stem cells, gene transfer, *ex vivo* therapy, intravenous transplantation, neuroprotection

## Abstract

Hepatocyte growth factor (HGF) has neuroprotective effects against ischemia-induced injuries. Dental pulp stem cell (DPSC) transplantation attenuates tissue injury in the brain of rats with post-transient middle cerebral artery occlusion. We sought to determine whether DPSCs that overexpress HGF can enhance their therapeutic effects on brain damage post-ischemia/reperfusion injury. Treatment with DPSCs overexpressing HGF reduced infarct volumes compared to unmodified DPSC treatment at 3 and 7 days post-transient middle cerebral artery occlusion. The use of unmodified DPSCs and DPSCs overexpressing HGF was associated with improved motor function compared to that with administration of vehicle at 7 days post-transient middle cerebral artery occlusion. DPSCs overexpressing HGF significantly inhibited microglial activation and pro-inflammatory cytokine production along with suppression of neuronal degeneration. Post-reperfusion, DPSCs overexpressing HGF attenuated the decreases in tight junction proteins, maintained blood-brain barrier integrity, and increased microvessel density in peri-infarct areas. The administration of DPSCs overexpressing HGF during the acute phase of stroke increased their neuroprotective effects by modulating inflammation and blood-brain barrier permeability, thereby promoting improvements in post-ischemia/reperfusion brain injury.

## Introduction

Ischemic stroke is a leading cause of death and severe neurological disabilities worldwide. Recombinant tissue plasminogen activator and endovascular therapies are clinically effective in acute ischemic stroke, but they have narrow therapeutic windows and eligibility criteria, and only a few patients can undergo recanalization therapies.^1^, ^2^ Therefore, new treatments for ischemic stroke are needed.

Cell-based therapies, including the transplantation of bone marrow-derived (BM) mesenchymal stem cells (MSCs), adipose tissue-derived MSCs, human umbilical cord blood cells, and neural stem cells, restore neurological function after ischemic stroke.^3^, ^4^, ^5^, ^6^, ^7^, ^8^ Dental pulp stem cells (DPSCs) are neural crest-derived stem cells that are an appealing cell source because they can be easily obtained as medical waste without ethical or logistic problems.^9^, ^10^ Compared to BM-MSCs, DPSC isolation is less invasive, and DPSCs are more easily expanded and they have stronger immunosuppressive properties.^10^, ^11^, ^12^ The neurotrophic factors secreted by DPSCs, for example, nerve growth factor, neurotrophin-3, brain-derived neurotrophic factor, and vascular endothelial growth factor (VEGF), promote neuronal survival, proliferation, differentiation, and migration.^13^, ^14^, ^15^, ^16^ Several studies have shown that DPSC transplantation has neuroprotective effects and enhances functional recovery after cerebral ischemia *in vivo*.^17^, ^18^

Hepatocyte growth factor (HGF) was initially purified and identified as a potent hepatocyte mitogen.^19^, ^20^ HGF is a powerful pleiotropic cytokine that induces angiogenesis, mitosis, and tissue regeneration, as well as anti-apoptotic and anti-inflammatory effects in a variety of organs.^21^, ^22^ The therapeutic effects of HGF in several disease models have been described *in vivo*, and clinical trials investigating HGF therapy for peripheral arterial disease, coronary artery disease, spinal cord injuries, and amyotrophic lateral sclerosis are ongoing.^23^, ^24^, ^25^, ^26^, ^27^ Furthermore, it was demonstrated that HGF gene administration might inhibit blood-brain barrier (BBB) disruption and exhibit neuroprotective effects after cerebral ischemia *in vivo*.^28^ Therefore, we hypothesized that the combination of *ex vivo* HGF gene therapy and DPSCs would attenuate inflammatory responses and BBB disruption after cerebral ischemia.

In this study, we intravenously transplanted DPSCs overexpressing the HGF gene in rats during the acute phase of stroke to enhance the therapeutic effects of DPSCs in brain damage after ischemia/reperfusion (I/R) injury.

## Results

### Hepatocyte Growth Factor Expression Levels from DPSCs

**Figure 1:**
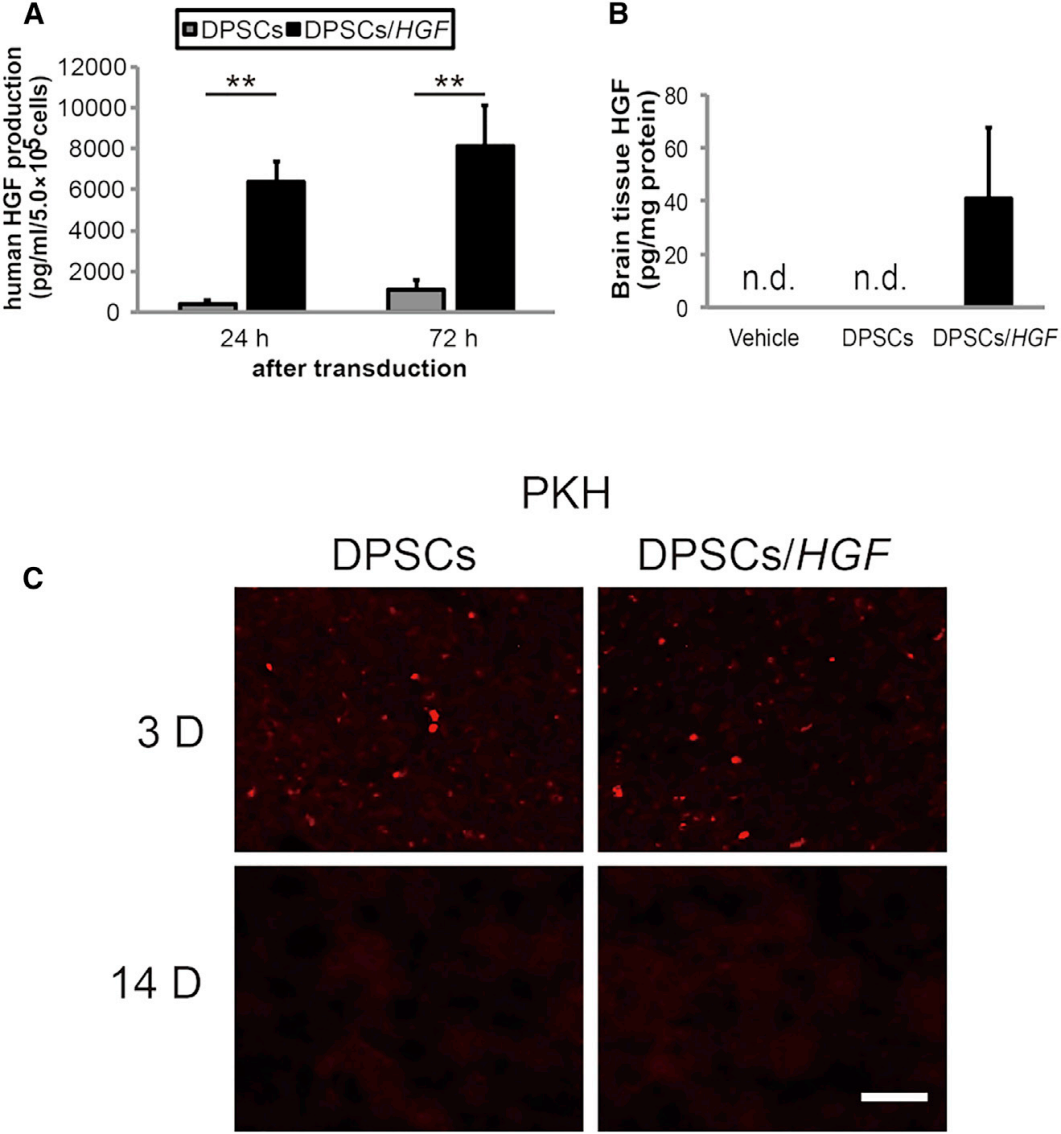
Quantification of Hepatocyte Growth Factor Expression in the Culture Media and Ischemic Hemispheres and Tracking of Engrafted DPSCs (A) DPSCs were transduced with a recombinant self-complementary adeno-associated virus vector that harbored the cytomegalovirus promoter-driven hepatocyte growth factor (HGF) expression cassette. The HGF concentrations were determined in the media of DPSCs or HGF-transduced DPSCs cultured at 5 × 10^5^ cells per mL for 24 hr or 72 hr (n = 3/group). The data presented are the means and the SDs (**p < 0.01). (B) ELISA quantification of HGF expression in the ischemic hemispheres 72 hr after tMCAO (n = 3/group). (C) Tracking of the engrafted DPSCs and DPSCs/*HGF* in the ischemic hemispheres using PKH26 labeling 3 and 14 days post-transient middle cerebral artery occlusion (tMCAO). Fluorescent microscopy detected red fluorescent engrafted cells. The scale bar represents 100 μm.

We used ELISAs to measure HGF concentrations in the culture media of unmodified and *HGF* gene-transduced DPSCs (DPSCs/*HGF*) (Figure 1A). Twenty-four hours after transduction, the mean (±SD) concentration of HGF protein was 395.5 ± 173.5 pg/mL in unmodified DPSCs and 6,366.6 ± 998.4 pg/mL in DPSCs/*HGF* (p < 0.01). Seventy-two hours after transduction, these concentrations increased to 1,098.8 ± 447 pg/mL and 8,123 ± 1,976.6 pg/mL (p < 0.01), respectively.

### Hepatocyte Growth Factor Expression in the Ischemic Brain and Serum

This study involved three experimental groups: DPSCs, i.e., transplanted with unmodified DPSCs after transient middle cerebral artery occlusion (tMCAO); DPSCs/*HGF*, i.e., transplanted with HGF gene-transduced DPSCs after tMCAO; and vehicle, i.e., received PBS after tMCAO. Sham-operated animals were subjected to the same surgical procedures as in the experimental groups, but without sutures for intraluminal occlusion. We evaluated the HGF levels in brain tissue extracts and serum samples at 72 hr post-tMCAO. HGF was detected in the brain tissue extracts from DPSCs/*HGF*-treated rats (Figure 1B), but not in serum samples (data not shown).

### Tracking the Engrafted DPSCs

The unmodified DPSCs and DPSCs/*HGF* that were labeled with PKH26 before transplantation were detected in the ischemic hemispheres. PKH26-labeled cells were detected in the DPSC and DPSC/*HGF* groups at 3 days post-tMCAO, but not at 14 days post-tMCAO (Figure 1C).

### Cerebral Infarct Volumes and Neurological Symptoms

**Figure 2:**
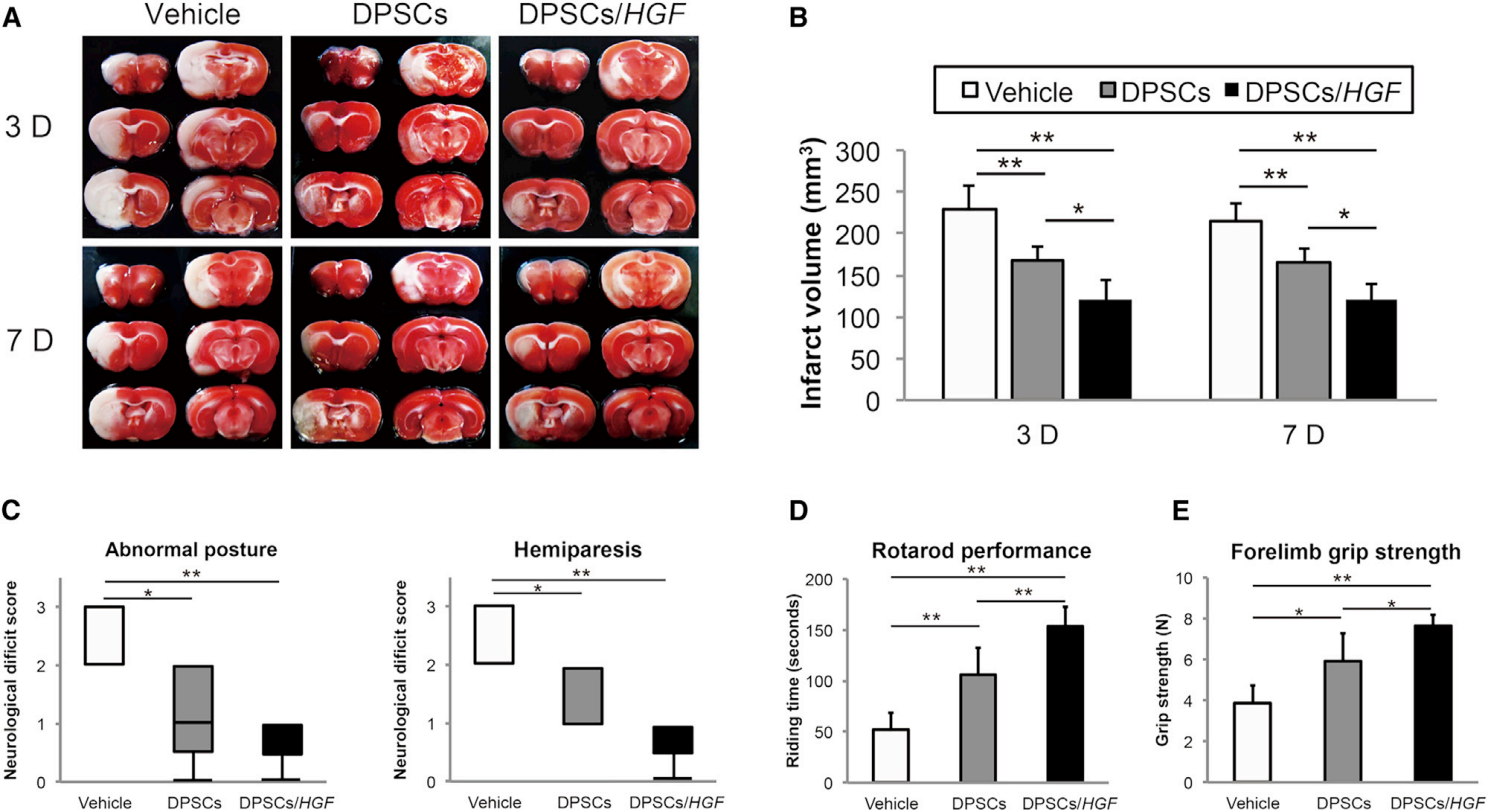
Reduction of the Infarct Volumes Was Detected Using Triphenyltetrazolium Chloride Staining and Improvements in Neurobehavioral Outcomes (A) Triphenyltetrazolium chloride (TTC) staining 3 days and 7 days after transient middle cerebral artery occlusion (tMCAO) in the vehicle, dental pulp stem cells (DPSCs)-, or DPSCs/hepatocyte growth factor gene (DPSCs/*HGF*)-treated groups (n = 5/group). (B) Quantitative analysis of the infarct volumes 3 days and 7 days post-tMCAO in each group (n = 5/group). The data presented are the means and the SDs (*p < 0.05, **p < 0.01). (C) Abnormal posture and hemiparesis assessments 7 days post-tMCAO in the vehicle-, DPSCs-, or DPSCs/*HGF*-treated groups (n = 5/group). The boxplots indicate the medians and interquartile ranges, and the whiskers indicate the maximum and minimum values (*p < 0.05, **p < 0.01). (D) Rotarod performances and (E) forelimb grip strength 7 days post-tMCAO in the vehicle-, DPSCs-, and DPSCs/*HGF*-treated groups (n = 5/ group) (*p < 0.05, **p < 0.01).

The infarct volumes were determined from 2,3,5-triphenyltetrazolium chloride (TTC)-stained sections 3 and 7 days after tMCAO (Figure 2A). The mean (±SD) infarct volumes 3 days post-tMCAO were significantly lower in the DPSC (167.4 ± 17.9 mm^3^, p < 0.01) and DPSC/*HGF* (119.5 ± 25.4 mm^3^, p < 0.01) groups compared to those in the vehicle group (229.8 ± 26.7 mm^3^) (Figure 2B). The infarct volume in the DPSCs/*HGF* was significantly lower than that in the DPSC (p < 0.05) group. Seven days post-tMCAO, the mean (±SD) infarct volume in the DPSC (165 ± 16.8 mm^3^) and DPSC/*HGF* (121.1 ± 22.3 mm^3^) groups had declined significantly compared to that in the vehicle group (215.3 ± 20.6 mm^3^) (both p < 0.01) (Figure 2B). DPSC/*HGF* treatment led to a greater reduction in the infarct volume compared to that achieved with DPSC treatment (p < 0.05).

We evaluated the neurological scores and motor function recovery 7 days post-tMCAO. The rats in the DPSC and DPSC/*HGF* groups showed significant preservation of posture (DPSCs, p < 0.05; DPSCs/*HGF*, p < 0.01) and palsy score (DPSCs, p < 0.05; DPSCs/*HGF*, p < 0.01) compared to those in the vehicle group (Figure 2C). In addition, rats in the DPSC and DPSC/*HGF* groups showed significant improvements in their rotarod performances (DPSCs, p < 0.01; DPSCs/*HGF*, p < 0.01) (Figure 2D) and forelimb grip strengths (DPSCs, p < 0.05; DPSCs/*HGF*, p < 0.01) (Figure 2E) compared to those in the vehicle group.

### Microglial Activation

**Figure 3:**
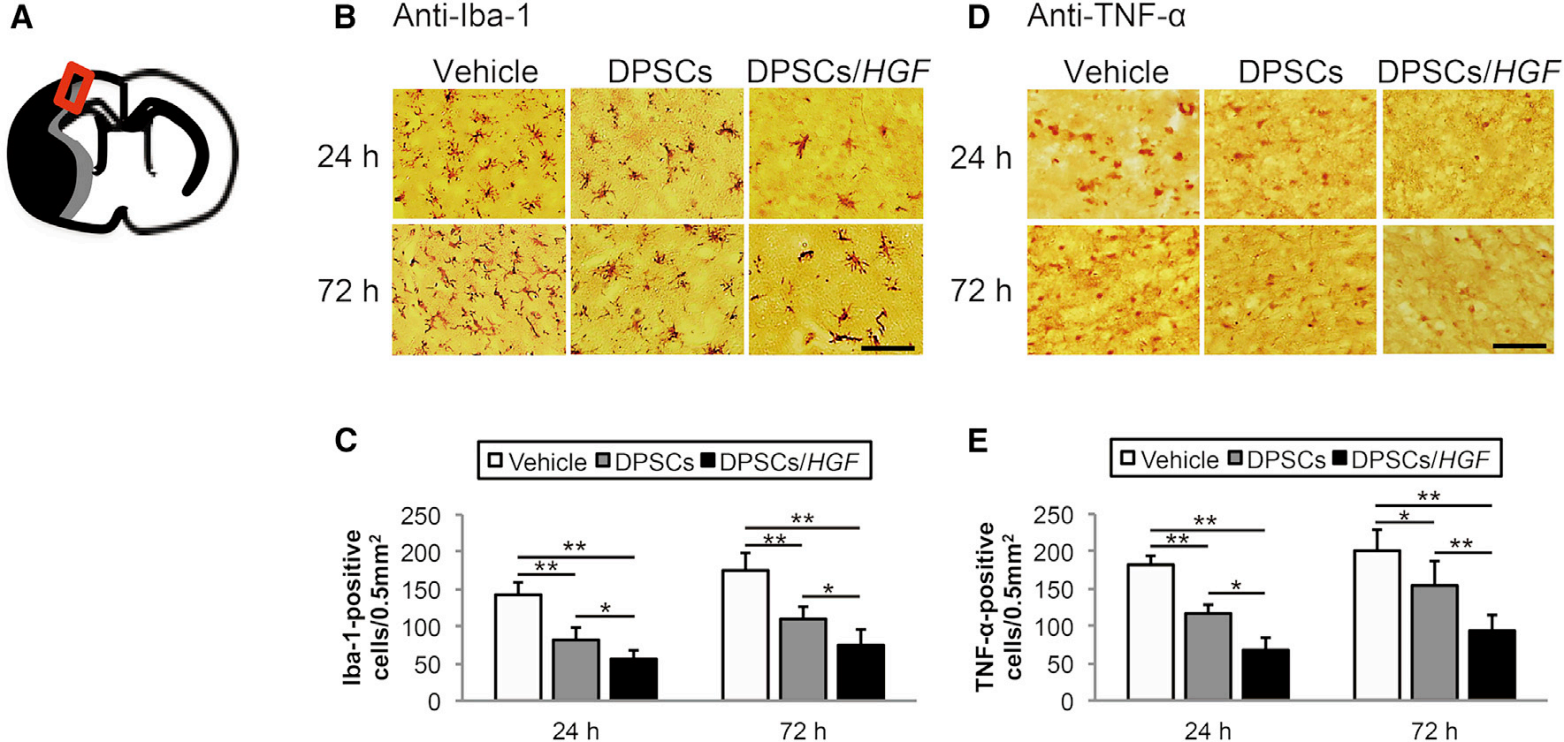
Immunohistochemical Staining for Ionized Calcium-Binding Adaptor Molecule and Tumor Necrosis Factor Alpha in the Cortical Ischemic Boundary Zone (A) The rectangle with the red outline on the diagram indicates the cortical ischemic boundary zone. The black area represents the ischemic lesion. (B) Ionized calcium-binding adaptor molecule (Iba-1) staining 24 hr and 72 hr post-transient middle cerebral artery occlusion (tMCAO) (n = 5/group). The scale bar represents 100 μm. (C) Numbers of immunopositive cells. The data presented are the means and the SDs (*p < 0.05, **p < 0.01). (D) Tumor necrosis factor alpha (TNF-α) staining 24 hr and 72 hr post-tMCAO (n = 5/group). The scale bar represents 100 μm. (E) Numbers of immunopositive cells. The data presented are the means and the SDs (*p < 0.05, **p < 0.01).

Ionized calcium-binding adaptor molecule 1 (Iba-1) is a microglia-specific calcium-binding protein and Iba-1 expression is associated with microglia activated by ischemic brain injury. We analyzed the localization of Iba-1 in the cerebral cortex at the ischemic border zone (IBZ) to assess microglial activation after DPSCtransplantation (Figure 3B). The numbers of Iba-1-positive cells in the DPSC and DPSC/*HGF* groups were significantly lower than those in the vehicle group at 24 hr (DPSCs, p < 0.01; DPSCs/*HGF*, p < 0.01) and 72 hr (DPSCs, p < 0.01; DPSCs/*HGF*, p < 0.01) post-tMCAO (Figure 3C). Furthermore, the numbers of Iba-1-positive cells were significantly lower in the DPSC/*HGF* group compared to those in the DPSC group at 24 hr (p < 0.05) and 72 hr (p < 0.05) after reperfusion.

### Pro-inflammatory Cytokine Levels

Immunohistochemical staining of the pro-in flammatory cytokine tumor necrosis factor alpha (TNF-α) was performed using an anti-TNF-α antibody (Figure 3D). The numbers of TNF-α-positive cells in the DPSC and DPSC/*HGF* groups were significantly lower than those in the vehicle group at 24 hr (DPSCs, p < 0.01; DPSCs/*HGF*, p < 0.01) and 72 hr (DPSCs, p < 0.05; DPSCs/*HGF*, p < 0.01) post-tMCAO (Figure 3E). Compared to the DPSC group, the numbers of TNF-α-positive cells in the DPSC/*HGF* group were significantly lower at 24 h (p < 0.05) and 72 hr (p < 0.01) post-tMCAO.

**Figure 4:**
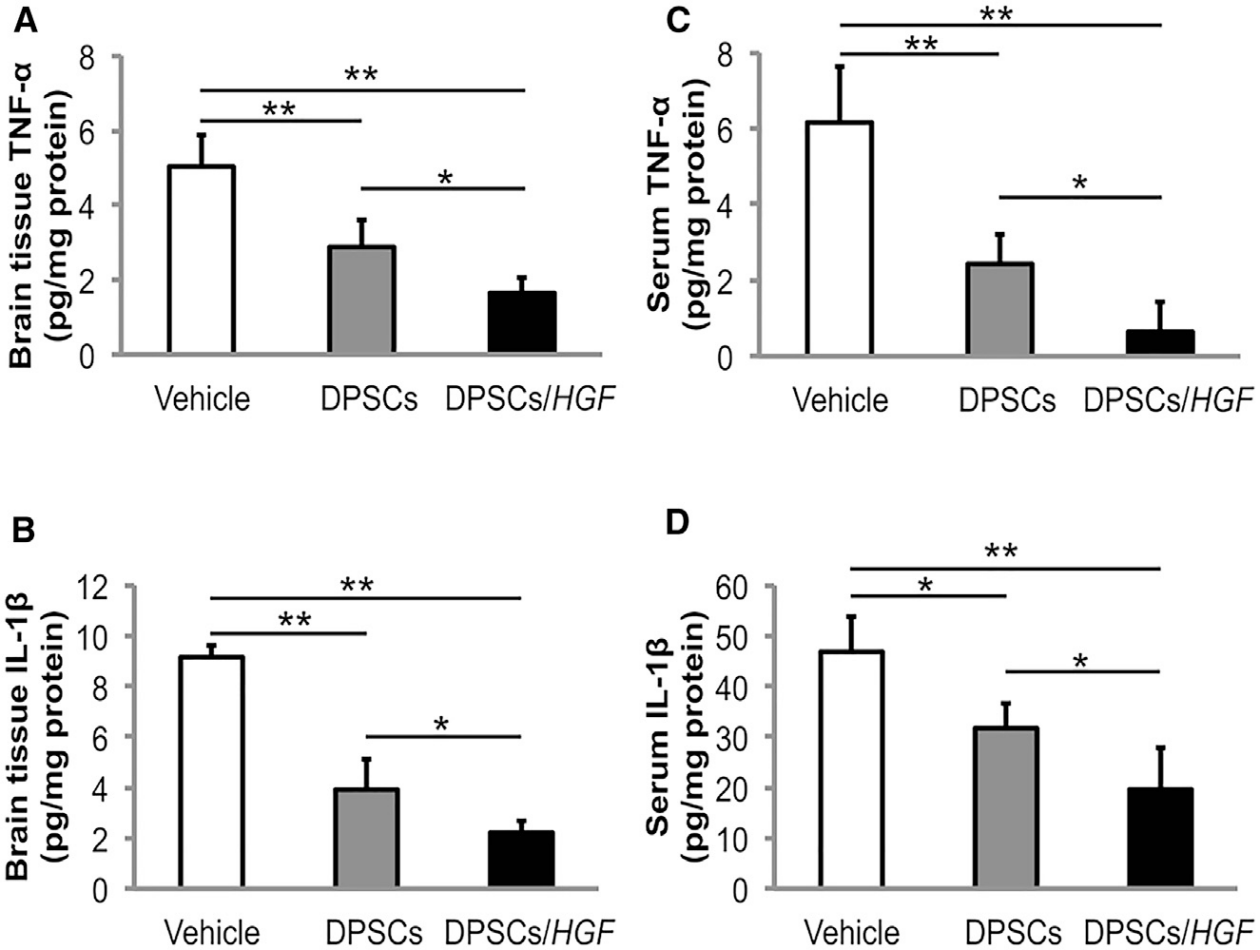
The Effect of Dental Pulp Stem Cell Transplantation on the Expression of Tumor Necrosis Factor Alpha and Interleukin-1β in the Brain Tissue Extracts or Serum Samples using Enzyme-Linked Immunosorbent Assays Quantification of tumor necrosis factor alpha (TNF-α) expression in the (A) ischemic hemisphere extracts and (C) serum at 72 hr post-transient middle cerebral artery occlusion (tMCAO) (n = 5/group). Quantification of interleukin (IL)-1β expression in (B) ischemic hemisphere extracts and (D) serum at 72 hr post-tMCAO (n = 5/group). The data presented are the means and the SDs (*p < 0.05, **p < 0.01).

The TNF-α and interleukin (IL)-1β concentrations in brain tissue extracts and serum were determined using enzyme-linked immunosorbent assays at 72 hr post-tMCAO. The brain tissue extracts from rats in the DPSC/*HGF* group contained significantly lower TNF-α and IL-1β levels than those in the vehicle (TNF-α, p < 0.01; IL-1β, p < 0.01) and DPSC groups (TNF-α, p < 0.05; IL-1β, p < 0.05) (Figures 4A and 4B). In addition, rats in the DPSC/*HGF* group had significantly lower TNF-α and IL-1β levels in their serum samples than those in the vehicle (TNF-α, p < 0.01; IL-1β, p < 0.01) and DPSC group rats (TNF-α, p < 0.05; IL-1β, p < 0.05) (Figures 4C and 4D).

### Ischemia-Induced Neuronal Damage

**Figure 5:**
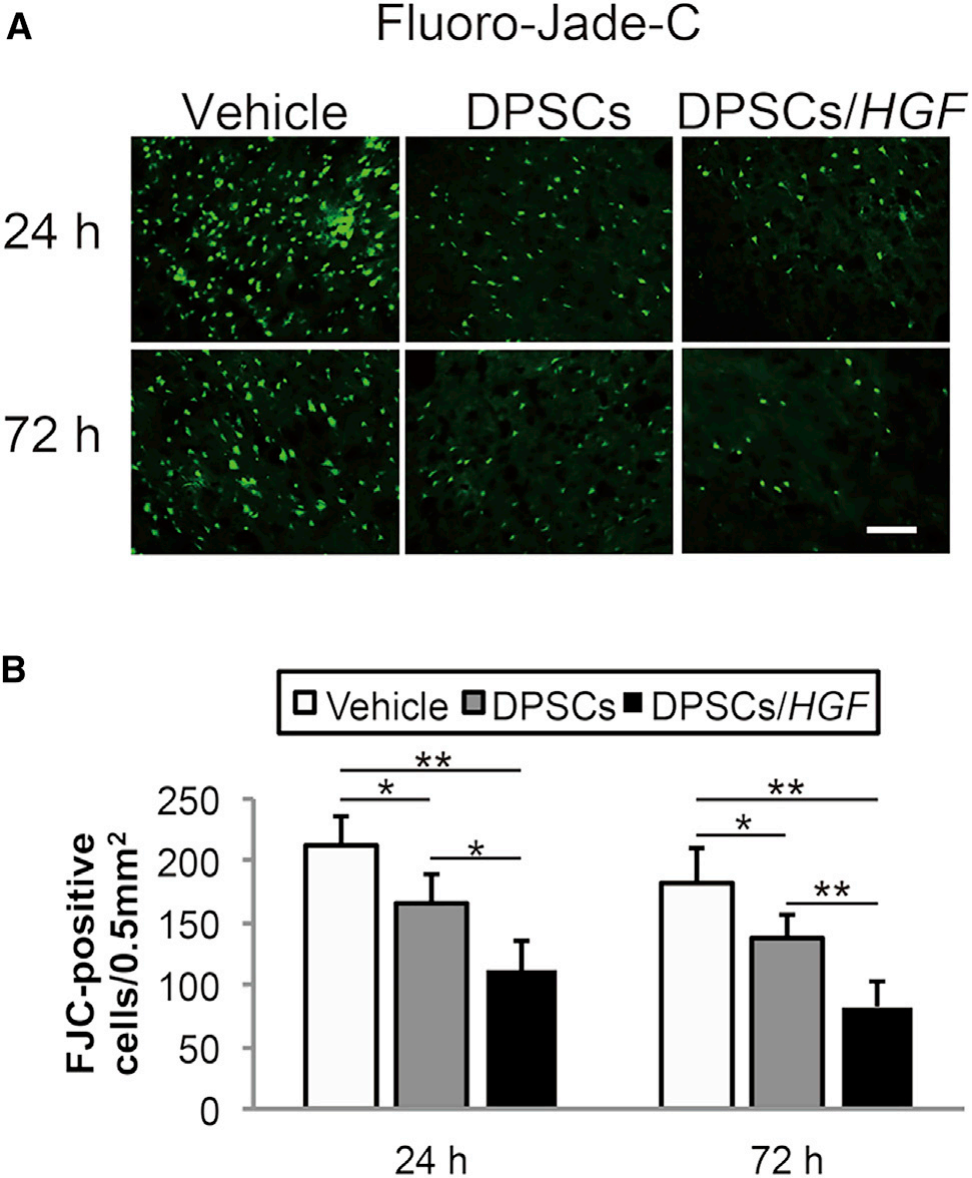
Fluoro-Jade C Staining of Neuronal Degeneration in the Cortical Ischemic Boundary Zone (A) Fluoro-Jade C staining at 24 hr and 72 hr post-transient middle cerebral artery occlusion. The scale bar represents 100 μm. (B) Numbers of immunopositive cells. The data presented are the means and the SDs (*p < 0.05, **p < 0.01) (n = 5/group).

To evaluate the effect on neuronal degeneration, we performed Fluoro-Jade C (FJC) staining in the cortical IBZ (Figure 5A). The numbers of FJC-positive cells in the DPSC and DPSC/*HGF* groups were significantly lower than those in the vehicle groups at 24 hr (DPSCs, p < 0.05; DPSCs/*HGF*, p < 0.01) and 72 hr (DPSCs, p < 0.05; DPSCs/*HGF*, p < 0.01) post-tMCAO (Figure 5B). In addition, the numbers of FJC-positive cells were markedly lower in the DPSC/*HGF* group compared to those in the DPSC group at 24 hr (p < 0.05) and 72 hr (p < 0.01) post-tMCAO.

### Evans Blue Extravasation

**Figure 6:**
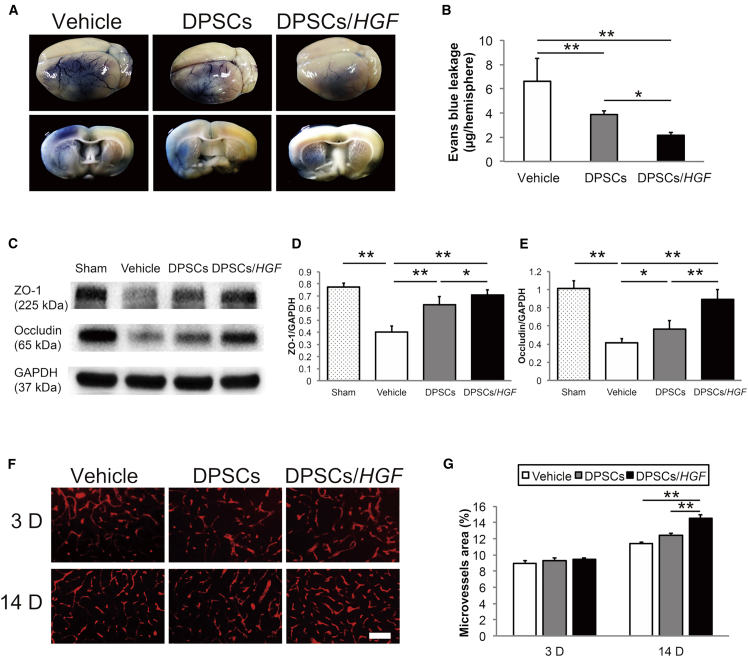
Effect of Hepatocyte Growth Factor Gene Modification on the Blood-Brain Barrier (A) Evans Blue extravasation in the brain and coronal sections after vehicle, dental pulp stem cell (DPSC), or DPSC/*HGF* treatment at 72 hr post-tMCAO. (B) Quantitative evaluation of Evans Blue leakage in the ischemic hemispheres 72 hr post-transient middle cerebral artery occlusion (tMCAO) (n = 6/group). The data presented are the means and the SDs (*p < 0.05, **p < 0.01). (C) Western blot analysis of the tight junction proteins in the ischemic hemispheres at 72 hr post-tMCAO (n = 7/group). (D) The ratios of ZO-1 and (E) occludin to glyceraldehyde 3-phosphate dehydrogenase (GAPDH), respectively. The data presented are the means and the SDs (*p < 0.05, **p < 0.01). (F) RECA-1 staining of the microvessels in the cortical ischemic boundary zone at 3 days and 14 days post-tMCAO (n = 6/group). The scale bar represents 100 μm. (G) Quantification of the microvessel density at 3 days and 14 days post-tMCAO. The data presented are the means and SDs (**p < 0.01).

We examined BBB leakages 72 hr post-tMCAO (Figure 6A). The BBB leakages were significantly reduced in the DPSCs- (p < 0.01) and DPSCs/*HGF*-treated rats (p < 0.01) compared to those in the vehicle group (Figure 6B). The rats in the DPSC/*HGF* group showed greater BBB leakage reduction compared to rats in the DPSCs-treated group (p < 0.05).

### Tight Junction Protein Protection

The tight junction protein levels in the ischemic hemispheres 3 days post-tMCAO were evaluated using western blots (Figure 6C). A significant increase in the level of ZO-1 expression was seen in the DPSCs/*HGF*-treated rats compared to those in the vehicle (p < 0.01) and DPSC groups (p < 0.05) (Figure 6D). The level of occludin expression was significantly higher in the DPSC/*HGF* group compared to that in the vehicle (p < 0.01) and DPSC groups (p < 0.01) (Figure 6E).

### Angiogenesis in the Cortical IBZ

We evaluated the densities of microvessels in the cortical IBZ using an anti-RECA-1 antibody, which is an endothelial cell marker, 3 and 14 days post-tMCAO (Figures 6F and 6G). The microvessel density was significantly higher in the DPSC/*HGF* group compared to that in the vehicle (p < 0.01) and DPSC groups (p < 0.01) at 14 days post-tMCAO. Microvessel density at 3 days post-tMCAO was not significantly different among the groups.

## Discussion

The present study demonstrated that after tMCAO, DPSC/*HGF* treatment is more effective than unmodified DPSC treatment, as indicated by the attenuation of brain damage and the improvement in neurological recovery. DPSC/*HGF* treatment suppressed pro-inflammatory cytokine levels in the ischemic hemispheres and ameliorated the disruption of BBB by inhibiting the decline in tight junction proteins, which further reduced the level of subsequent ischemia-induced neuronal damage and promoted angiogenesis after cerebral I/R injury.

Cerebral ischemia provokes inflammatory responses, characterized by rapid microglial activation, pro-inflammatory cytokine production, and infiltration of inflammatory cells into the injured brain tissue.^29^ These responses play pivotal roles in infarct progression and edema formation after an I/R brain injury.^30^ Functional roles for TNF-α, which is a pro-inflammatory cytokine, have been demonstrated in several I/R models, including those of I/R brain injury.^31^ IL-1β is another pro-inflammatory cytokine that is expressed in response to I/R injury, increasing its severity.^32^ Since inflammatory cytokines from the activated microglia are upregulated in the brain at a relatively late stage after brain injury, we investigated inflammatory responses in the brain and plasma at 1–3 days after reperfusion.^33^

DPSC/*HGF* treatment suppressed the levels of TNF-α and IL-1β expression in the ischemic brain tissue and serum, and the level of microglial activation in the ischemic brain tissue to greater extents compared to those achieved by unmodified DPSC treatment. Furthermore, DPSCs/*HGF* significantly reduced the level of neuronal damage in the cortical IBZ. Hence, DPSC/*HGF* treatment might enhance the neuroprotective effects of DPSCtransplantation by modulating the effects of pro-inflammatory cytokines on I/R brain injury.

BBB disruption initiates events that lead to vasogenic edema following brain injury. Recent studies have shown that HGF inhibits the decline in tight junction protein levels and reduces the disruption of BBB, thereby attenuating the brain edema that follows an ischemic stroke.^28^, ^34^, ^35^, ^36^ As expected, our results showed that HGF prevented declines in the occludin and ZO-1 levels and maintained integrity of BBB in the ischemic hemisphere at 3 days after I/R brain injury. These results suggest that DPSCs/*HGF* enhances the protective effects of DPSC transplantation at the level of tight junction proteins and reduces the disruption of BBB during the acute phase of cerebral ischemia.

Angiogenesis takes place in stroke-affected regions and is essential for ischemic brain repair, because it stimulates blood flow and metabolism in the IBZ.^37^ VEGF, which is one of the most studied growth factors, exerts remarkable pro-angiogenic and vascular permeability effects after stroke that are necessary for the formation of new vessels by angiogenesis.^38^ VEGF is also secreted by DPSCs; hence, these cells can release angiogenic factors extracellularly.^13^, ^39^ Transplanted cells promote the secretion of angiogenic cytokines, which increase the proliferation of existing vascular endothelial cells during the first 14 days after cerebral ischemia.^40^, ^41^ We have shown that the numbers of RECA-1-positive cells in the cortical IBZ increased compared to those in the control group and that DPSC/*HGF* treatment significantly increased microvessel density in the IBZ at 14 days after reperfusion compared to that with unmodified DPSC treatment. In addition, HGF itself promotes angiogenesis after cerebral ischemia.^42^ The overexpression of HGF may augment the synergistic effects of HGF and VEGF. Further studies are needed to investigate how HGF affects the expression of tight junction proteins and angiogenesis after cerebral ischemia.

Both the engrafted DPSCs/*HGF* and the non-genetically modified DPSCs were detected in the ischemic hemispheres at 3 days but disappeared by 14 days post-tMCAO. In addition, we determined that DPSC/*HGF* treatment increased HGF levels in the ischemic brain without affecting serum HGF levels. While HGF provokes a variety of cellular signals that mediate tumor growth and metastasis, DPSCs/*HGF* may reduce the risk of HGF-induced adverse reactions, because the engrafted DPSCs survive for a short time only and the elevation of HGF levels in the ischemic brain are localized.^43^

The AAV vector is derived from a small parvovirus and appears to be promising for human gene therapy. Compared to other viral vectors, including adenovirus, the host immune response to AAV vectors is mild.^44^ Retrovirus and lentivirus have relatively high transduction efficiencies, but random integration can occur that disrupts gene function or promotes oncogenic development. The AAV vector causes insertional mutagenesis at a very low frequency.^45^ In this study, we transduced the DPSCs with AAV, which we consider a safer therapeutic approach than administering the AAV vector directly.

Although this study demonstrated the enhanced protective effects of DPSC/*HGF* treatment against I/R injury, some study limitations should be acknowledged, which include the short observation period and the failure to investigate this therapy’s therapeutic window. We observed the effects of DPSCs/*HGF* only during transplantation, which occurred immediately after reperfusion. More fundamental studies are needed to elucidate the optimal therapeutic window and any long-term effects of DPSC/*HGF* transplantation for the treatment of stroke.

In conclusion, treatment with HGF-overexpressing DPSCs attenuated the inflammatory reaction and BBB disruption after I/R brain injury, which appeared to enhance the neuroprotective effects of DPSCtransplantation and were perhaps related to ischemic brain repair during the acute phase of cerebral ischemia.

## Materials and Methods

### Cell Preparation

The human DPSCs were provided by JCR Pharmaceuticals (Ashiya, Hyōgo Prefecture, Japan). The cells were cultured in DMEM (Thermo Fisher Scientific, Waltham, MA), supplemented with 10% fetal bovine serum (Thermo Fisher Scientific), 1% antibiotic-antimycotic solution (Wako Pure Chemical Industries, Osaka, Japan), and 50 μg ascorbic acid 2-phosphate (Wako Pure Chemical Industries) at 37°C in an atmosphere of 98% humidity and 5% CO_2_. Cell viability was determined using the trypan blue dye exclusion method.

### Plasmid and Vector Construction

A self-complementary (sc) AAV vector pro-viral genome that harbored the cytomegalovirus (CMV) promoter, human HGF cDNA, and the simian virus (SV)40 polyadenylation signal sequence, was fully synthesized, cloned into the pUC57 backbone as a vector pro-viral plasmid, and verified using Sanger sequencing at GenScript (Piscataway, NJ). A recombinant scAAV1 vector (scAAV1-CMV-HGF) was obtained in a serum-free culture medium using polyethylenimine-based triple transfections of HEK293 cells.^46^ The viral titer was determined using real-time PCR (7500 Fast Real-Time PCR System; Applied Biosystems, Foster City, CA), with the appropriate forward (5′-TAAGCTCCAAGACAAAGGTG-3′) and reverse (5′-GTCCTGCAGTCCAGTAGATG-3′) primers.

### *Ex Vivo* Gene Delivery to the DPSCs

Cultured DPSCs (1.0 × 10^6^ cells) from the seventh to ninth passages were infected with the virus suspension at a concentration of 1 × 10^5^ vector genomes per cell, and they were incubated in serum-free media for 24 hr before transplantation.

### tMCAO Animal Model

All of the animal procedures were performed per the guidelines established by the Animal Committee at the Graduate School of Nippon Medical School. Eight-week-old male Sprague-Dawley rats that weighed 250–300 g were purchased from Sankyo Labo Service (Tokyo, Japan). The animals were anesthetized with 5% halothane, and anesthesia was maintained using 0.5%–1.0% halothane in a 70% N_2_O and 30% O_2_ mixture, while the animals breathed spontaneously.

A total of 151 rats were subjected to transient cerebral focal ischemia for 90 min using an intraluminal suture method.^47^ In brief, the left common and internal and external carotid arteries were exposed through a midline cervical incision. A 4-0 nylon thread with a silicone-coated tip was inserted into the left internal carotid artery and up to the origin of the left middle cerebral artery. After arterial occlusion for 90 min, the nylon thread was withdrawn to allow reperfusion. A PE-50 catheter was inserted into the tail artery for blood sampling and to monitor the blood pressure. The mean (±SD) temporal muscle temperature was maintained at 37°C ± 0.5°C during the operation. 135 rats were successfully subjected to 90 min-MCAO, and 16 rats were excluded after applying the following criteria: inappropriate occlusion of the MCA (n = 11) or sudden death during the surgery process (n = 5).

### Intravenous DPSC Transplantation

A PE-50 catheter was inserted into each animal’s tail vein. The study comprised three experimental groups that received treatments as described next. Unmodified DPSCs (1 × 10^6^ cells in 1 mL of PBS), DPSCs/*HGF* (1 × 10^6^ cells in 1 mL of PBS), or the vehicle (1 mL of PBS) were intravenously transplanted into each animal through the catheter immediately after tMCAO. None of the animals received immunosuppressive drugs.

### PKH26 Labeling and Immunofluorescent Staining

A PKH26 red fluorescent cell linker kit (Sigma-Aldrich, St. Louis, MO) was used to track the transplanted unmodified DPSCs and DPSCs/*HGF* in the brain. The animals were injected with these labeled DPSCs and were decapitated 3 or 14 days after tMCAO. The animals were perfused with heparinized saline. Coronal sections were cut at 20-μm intervals using a cryostat, and the sections were mounted using Vectashield with DAPI (Vector Laboratories). The fluorescent images were captured at a 200× magnification using a Nikon Eclipse E500W microscope (Nikon Corporation, Minato, Tokyo, Japan).

### Infarct Volumes

The animals were anesthetized and decapitated 3 or 7 days after tMCAO to analyze the infarct volumes. Each brain was cut into coronal sections at 2-mm intervals and stained with TTC (Wako Pure Chemical Industries) for 20 min at 37°C in the dark. Blinded personnel traced the infarcted areas using the ImageJ software (NIH, Bethesda, MD). The infarct areas in each slice were added separately and multiplied by the slice thickness to obtain infarct volumes (mm^3^).

### Neurological Symptoms

The neurological symptoms were assessed 7 days after tMCAO by an observer who was blinded to the study’s protocol, using a scoring system that was designed to evaluate hemiparesis and abnormal posture.^48^ The right hindlimb of each animal was extended using round-tipped forceps, and the flexor response was scored as follows: 0, normal; 1, slight deficit; 2, moderate deficit; or 3, severe deficit. It was suspended by the tail, and the forelimb flexion and body twisting were scored to assess the animal’s posture as follows: 0, normal; 1, slight twisting; 2, marked twisting; or 3, marked twisting and forelimb flexion. Motor abnormalities were assessed using the rotarod performance test (Model 7750; Ugo Basile SRL, Varese, Italy). The rotarod moved at an initial speed of 4 rpm, and the rod’s speed was accelerated to 40 rpm within 300 s. The animals were habituated to the apparatus by repeating the test before recording their latency when falling off the rod. If an animal fell off the rod during the habituation session, it was placed back on the rod until it stayed for >150 s. Following the habituation period, the animal’s latency was recorded. Each animal underwent this test three times, and the average latency period was calculated. The strength of the forelimb grip was measured using a grip strength meter (Chatillon DFIS-10; Ametek Sensors, Test & Calibration, Largo, FL). The grip strength meter was horizontally positioned, and when the animals grasped a triangular pull bar with their forelimbs, they were pulled back on a horizontal plane. The maximum grip strength was measured in Newtons at the point when the grasp was released. The test was repeated on three consecutive occasions, and the average grip strength was calculated.

### Immunohistochemistry

The animals were anesthetized and perfused intracardially with heparinized saline followed by 4% paraformaldehyde 24 hr and 72 hr post-tMCAO. The brains were removed, fixed, and frozen rapidly. Frozen coronal sections were cut at 20-μm intervals using a cryostat. The sections were incubated with 0.3% hydrogen peroxidase in methanol for 30 min. After washing, non-specific binding was blocked with 10% goat serum in Tris-buffered saline. The sections were incubated overnight at 4°C with a rabbit polyclonal antibody against Iba-1 (Wako Pure Chemical Industries) that was diluted 1:500, or a rabbit polyclonal antibody against TNF-α (R&D Systems, Minneapolis, MN) that was diluted 1:100. Next, the sections were incubated with a biotinylated goat anti-polyvalent antibody (Thermo Fisher Scientific) at room temperature for 1 hr, followed by incubation with an avidin-biotin-peroxidase complex (Vector Laboratories, Burlingame, CA) for 30 min. The labeled secondary antibodies were visualized using diaminobenzidine. The positively labeled cells in the cortical IBZ were counted in three randomly chosen square fields of view (0.5 mm^2^) by an investigator who was blinded to the experimental groups. FJC staining was conducted using a commercially available kit (Fluoro-Jade C Ready-to-Dilute Staining Kit for Identifying Degenerating Neurons; Biosensis, Temecula, CA) according to the manufacturer’s protocol. An investigator who was blinded to the experimental groups counted the positively stained cells in an area (0.5 mm^2^) that was adjacent to the IBZ. Brain sections (20 μm) were incubated overnight at 4°C with a mouse monoclonal antibody against RECA-1 (Abcam, Cambridge, MA) that was diluted 1:300 to determine the densities of microvessels. The secondary antibody was an Alexa Fluor 568 goat anti-mouse antibody (Thermo Fisher Scientific) that was diluted 1:250. The samples were mounted using Vectashield with DAPI (Vector Laboratories). The microvessel densities in three randomly chosen square fields (0.5 mm^2^) in the cortical IBZ were determined using the ImageJ software (NIH).

### Enzyme-Linked Immunosorbent Assays

The HGF concentration in the cell culture medium, brain tissue extracts from the ischemic hemispheres, and serum samples were measured using a human HGF ELISA kit (R&D Systems) according to the manufacturer’s instructions. The concentrations of IL-1β and TNF-α in the brain tissue extracts and serum samples were measured using the appropriate ELISA kits (R&D Systems). Colorimetric absorbance was recorded at a wavelength of 450 nm.

### Evans Blue Extravasation

The disruption of the BBB was investigated 72 hr after tMCAO by measuring the extravasation of 2% Evans Blue dye (4 mL/kg) (Sigma-Aldrich Corporation) in normal saline that had been injected intravenously in the animals 1 hr before they were sacrificed. The animals were anesthetized and perfused with heparinized saline. The ipsilateral hemispheres were isolated and homogenized in 1 mL of N,N-dimethylformamide (Sigma-Aldrich), incubated at 55°C for 20 hr, and centrifuged at 20,000 × *g* for 20 min.^49^ The absorbance was measured at 620 nm with a spectrophotometer (Thermo Fisher Scientific).

### Western Blot Analysis

Western blots were performed to analyze the expression of tight junction proteins in the ischemic hemispheres. The proteins were extracted using radioimmunoprecipitation assay buffer. The sample proteins (50 μg) were separated using SDS-PAGE in 8%–14% gels (Bio-Rad Laboratories, Hercules, CA) with an SDS running buffer, and they were transferred onto polyvinylidene fluoride membranes overnight. The membranes were blocked with 3% BSA and Tris-buffered saline with Tween 20 for 2 hr at room temperature and incubated overnight at 4°C with primary antibodies to ZO-1 (Thermo Fisher Scientific, Carlsbad, CA) that was diluted 1:500, occludin (Thermo Fisher Scientific) that was diluted 1:500, or glyceraldehyde 3-phosphate dehydrogenase (GAPDH) (Cell Signaling Technology, Danvers, MA) that was diluted 1:5,000. After washing, the membranes were incubated with their respective horseradish peroxidase-labeled secondary antibodies, and the proteins were detected using the ECL Prime Western Blotting Detection Reagent (GE Healthcare, Little Chalfont, Buckinghamshire, UK). The band intensities were analyzed using ImageJ software (National Institutes of Health). All samples were normalized for protein loading by using GAPDH, which was loaded on the same membrane.

### Statistical Analyses

All data are presented as the means and SDs. Tukey’s honestly significant difference test was used to compare the infarct volumes, edema volumes, rotarod test balance times, forelimb grip strengths, immunohistochemical cell counts, Evans Blue dye extravasations, and the western blot and ELISA data. The Wilcoxon rank-sum test was used to compare the neurological scores. A value of p < 0.05 was considered statistically significant. JMP9 software (SAS Institute, Cary, NC) was used for all statistical analyses.

## Author Contributions

K.S. performed most of the experiments, wrote the manuscript, and contributed to the study concept and design. C.N. and T.O. conceived the experiments and wrote the paper. M.N. and A.N.-T. conceived and performed the experiments. S.S., Y.N., Y.S., Y.N.-K., M.U., and K.K. interpreted the data and supervised the project. All authors read and approved the manuscript.

## Conflicts of Interest

The authors have no conflicts of interest to declare.
